# High‐Performance Full‐Photolithographic Top‐Contact Conformable Organic Transistors for Soft Electronics

**DOI:** 10.1002/advs.202004050

**Published:** 2021-02-18

**Authors:** Xiaoli Zhao, Shuya Wang, Yanping Ni, Yanhong Tong, Qingxin Tang, Yichun Liu

**Affiliations:** ^1^ Center for Advanced Optoelectronic Functional Materials Research and Key Lab of UV‐Emitting Materials and Technology of Ministry of Education Northeast Normal University 5268 Renmin Street Changchun 130024 China

**Keywords:** conformable, organic transistors, photolithography, top‐contact geometry

## Abstract

Organic thin‐film transistors (OTFTs) are identified to be the most promising candidate for next‐generation wearable and implantable electronics because of their unique advantages including their flexibility, low cost, long‐term biocompatibility, and simple packaging. However, commercialization of organic transistors remains an enormous challenge due to their low mobility and lack of scalable strategy for high‐precise soft devices. Here, a novel photolithography fabrication strategy is proposed, which is completely compatible with various commercial organic semiconductor materials, for the first demonstration of the fully photolithographic top‐contact conformable OTFTs with the device density as high as 1523 transistors cm^−2^. Excellent electrical and mechanical properties with device yield as high as 100%, field‐effect mobility up to 1–2 cm^2^ V^−1^ s^−1^, and outstanding conformability are shown. This work provides a new strategy that can fully maximize the advantages of organic materials and photolithography technology, showing a great prospect in the development of high‐performance, high‐precise organic devices toward the commercialized and industrialized soft electronic products.

## Graphical abstract

A novel photolithography strategy that is completely “compatible with” various commercial organic semiconductor materials is proposed for the first demonstration of the fully photolithographic top‐contact organic thin‐film transistors (OTFTs). This strategy offers a general and facile route to incorporate photolithography technology into organic electronics, and presents potential for developing skin‐like soft electronic devices with high device density and high performance.

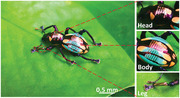

## 1 Introduction

Organic transistors, as one of the most basic building blocks of soft logic circuits and active matrix displays, have shown the unique application potential in next‐generation electronic products, such as electronic skin, flexible displays, implantable health monitors, and wearable sensors.^[^ ^1^, ^2^, ^3^, ^4^, ^5^, ^6^ ^]^ The motivations in developing organic transistors come from their mechanical flexibility, tunable electrical properties by molecular design, and low‐temperature, low‐cost processing.^[^ ^7^, ^8^, ^9^, ^10^, ^11^ ^]^ Currently, commercialization of organic transistors is challenging for two main reasons: low mobility and lack of scalable strategy for high‐precise soft devices. Photolithography, as the foundation of modern electronic and optoelectronic industry, has nowadays shown the irreplaceable advantage in large‐scale, high‐precise, and high‐integration production.^[^ ^12^, ^13^, ^14^, ^15^ ^]^ Compared with the other techniques such as shadow mask and printing, photolithography allows the feature size of devices to scale down to hundreds of nanometers with the extremely flexible and high‐precise pattern design. If organic transistors can be constructed completely by the photolithography technology, it will provide a vital breakthrough to propel future commercial electronic applications of organic electronics.

However, the intrinsic fragility of organic materials causes the organic semiconductor damage when exposed to solvents and UV light in photolithography process,^[^ ^4^ ^]^ resulting in the challenging issues in both organic semiconductor photolithography and electrode photolithography on organic semiconductor. Therefore, the currently reported photolithographic flexible organic transistors usually demonstrate the photolithography of either electrodes or semiconductor, or full‐photolithographic organic transistors based on a bottom‐gate, bottom‐contact geometry (Table S1, Supporting Information). The former faces the problem in low precision and/or crosstalk between neighboring devices, and the latter generally shows the low mobility because of the weaken gate‐field‐induced carrier injection. More importantly, the reported photolithography approaches are difficult to be fully compatible with flexible and elastic materials, which becomes the biggest obstacle to developing high‐integration and mass‐producible soft electronics.

Here, we develop a general strategy for full‐photolithographic top‐contact conformable organic transistors through the following ideas: 1) a photosensitive polyvinyl alcohol (PVA) layer is used to serve as both a protection layer and dielectric, which not only resolve the challenges in organic semiconductor photolithography, but also enable dielectric photolithography; 2) the photolithographic electrodes are separately prepared from semiconductor layer, which avoids the damage of the photolithographic electrodes on organic semiconductor; 3) a mechanical lamination process between electrodes and semiconductor is applied for bottom‐gate, top‐contact (BGTC) device geometry, which ensures the high mobility of the photolithographic organic transistors. In addition, the other striking point is that our photolithographic organic device is peeled off from the rigid substrate, showing the good compatibility with soft electronics. The resulting conformable transistor array is fabricated at near‐to‐room temperature (≤60 °C), and exhibits a high device density of 1523 transistors cm^−2^ and the mobility as high as 1–2 cm^2^ V^−1^ s^−1^. These key values are superior to state‐of‐the‐art photolithographic organic thin‐film transistors (OTFTs). The conformable OTFT array is extremely soft showing skin‐like conformability to arbitrary‐shaped objects, and at the same time maintains outstanding electrical performance. Our strategy offers a universal platform for constructing high‐performance, high‐integration OTFTs; maximizes the advantages of organic materials such as low‐temperature processing and mechanical flexibility; and demonstrates a strong potential in the development of next‐generation wearable and implantable electronics toward commercial flexible and elastic electronic products.

## 2 Results and Discussion

### 2.1 Device Design and Fabrication

**Figure 1:**
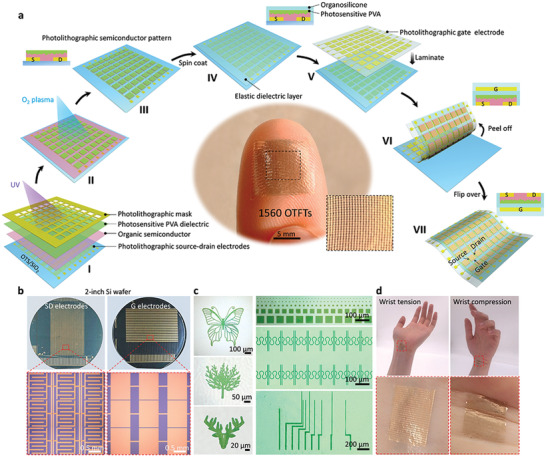
Platform for constructing the full‐photolithographic conformable organic transistor array. a) Fabrication scheme of the array: I) Organic semiconductor and photosensitive PVA were successively deposited on photolithographic source/drain electrodes, and then photosensitive PVA was patterned by photolithography. II,III) Based on the protection of the photopatterned photosensitive PVA, organic semiconductor was etched by oxygen plasma for forming photolithographic patterns; IV,V) elastic organosilicone was spin‐coated onto the sample, and then photolithographic gate electrode embedded in PDMS was laminated onto it; VI,VII) by peeling off the whole device from OTS/Si wafer, the full‐photolithographic conformable organic transistor array was obtained. The insets are the corresponding cross‐sectional diagrams and digital photos of a real array. An array of 1560 conformable transistors on a fingertip (middle insets), showing a device density of 1523 transistors per cm^2^. b) The array can be obtained at wafer scale. c) Transmission optical microscopy images of the obtained photolithographic conformable micropatterns. This versatile strategy can manufacture various sophisticated and high‐precision organic electronic devices. d) The array can well conform to the movement of human wrist.

**Figure** **1**a highlights the major fabrication scheme for full‐photolithographic conformable OTFTs array (see the “Methods” section and Figures S1 and S2 in the Supporting Information for details). This manufacturing strategy has enabled the realization of full‐photolithographic conformable OTFTs’ array. Initially, organic semiconductor and aqueous photosensitive PVA were successively deposited on photolithographic source–drain electrodes, and then photosensitive PVA crosslinking reaction was occurred under UV irradiation using a patterned chrome/quartz as mask, and following that fine photosensitive PVA patterns were formed after developing in water (Figure 1a‐ I,II). The photosensitive PVA in this process functions as photoresist, because it is water soluble and extremely mild without any damage to the organic semiconductor (Figure S3, Supporting Information), which differs from commonly used organic‐solvent‐based photoresist resulting in damage of the organic semiconductor.^[^ ^16^ ^]^ Next, based on the protection of photo‐crosslinked PVA, the patterned organic semiconductor can be nondestructively and precisely defined by oxygen plasma etching (Figure 1a‐ II,III). Subsequently, elastic organosilicone was spin‐coated onto the patterned sample (Figure 1a‐ III,IV). It is worth mentioning that both the photo‐crosslinked PVA and organosilicone function as the dielectric layer. Adding organosilicone layer, owing to its extraordinary dielectric properties, flexibility, and adhesion,^[^ ^17^ ^]^ can prevent electric leakage of devices and connect with subsequent gate electrode without any adhesive. Afterward, the photolithographic gate electrode embedded in polydimethylsiloxane (PDMS) was laminated on the organosilicone layer (Figure 1a ‐V). To further enhance the adhesion between them, we used oxygen plasma to pretreat their surface before laminating, and then utilized a low‐temperature postannealing process to form the strong bond after laminating. Finally, the whole OTFTs’ array was peeled off from octadecyltrichlorosilane (OTS)‐modified Si wafer, and was flipped over to form a full‐photolithographic conformable OTFTs’ array with a BGTC geometry (Figure 1a ‐VI,VII).

As is known to all, the formation of robust top contacts between electrodes and organic semiconductors is essential for the realization of high‐performance, large‐current‐density, high‐integration electronic devices. Top contact formation through directly depositing electrode materials on organic semiconductors has shown several drawbacks, such as the metal atoms penetrating or damaging the molecular layer.^[^ ^18^ ^]^ To avoid the drawbacks caused by the traditional vapor deposition technique, several top‐contact fabrication techniques have been developed. For instance, Vilan et al. used a “soft” method to avoid damaging the molecules,^[^ ^19^, ^20^ ^]^ and demonstrated that molecules can control the electrical characteristics of conventional metal–semiconductor junctions. Loo et al. introduced a novel approach, nanotransfer printing (nTP), to fabricate top‐contact electrodes in Au/1,8‐octanedithiol/GaAs junctions, that displayed more excellent photoelectric performance than conventional evaporation of Au electrode.^[^ ^21^ ^]^ Bonifas and McCreery reported a method of forming “soft” metallic contacts on molecular layers through surface‐diffusion‐mediated deposition, in which the metal atoms are deposited remotely and then diffuse onto the molecular layer, thus eliminating the problems of penetration and damage. Recently, Nawaz et al. presented a viable solution by applying rolled‐up metallic nanomembranes as the drain electrode, which enables the incorporation of a few nanometer thick semiconductor layers and usefully fabricated low‐voltage vertical organic transistors.^[^ ^22^ ^]^ Compared with previous reports, we applied the dry‐peeling strategy to form nondestructive top contact between metal electrode and semiconductor, which not only eliminates the problems of penetration and damage, but also can effectively be compatible with soft and conformable devices, showing the application potential in next‐generation wearable electronics.

Based on this new strategy, our fabricated transistor array presents an unprecedented device density of 1523 transistors cm^−2^, which is higher than that of all the reported conformable OTFTs.^[^ ^4^, ^23^ ^]^ Therefore, thousands of conformable devices can be integrated on a small fingertip as shown in the photograph in the middle panel of Figure 1. This novel strategy not only enables organic electronics to be compatible with the photolithography process, but also maximizes advantages of both organic electronics and photolithography technique, including wafer‐size production, high precision, small feature size, and good mechanical flexibility and conformability. It can be clearly seen in Figure 1b that the fabricated wafer‐scale device array is well defined with a sharp edge, and there are no residues in void areas (Figure S4, Supporting Information). Figure 1c also demonstrates that the versatile strategy can prepare various fine patterns, such as hollow butterfly, tree, deer, and serpentine curves. Moreover, their feature size can scale down to sub‐micrometer scale, which is extremely difficult to achieve with other technologies, such as shadow mask and printing. To further confirm the mechanical flexibility and conformability of our device array, we mounted it on human skin (Figure 1c). One edge of the device array contacts with the skin, and then van der Waals force causes the device array to gradually wet. The stamp process is reversible and nondestructive, and the device array can be easily put on and taken off without any damage. It can be obviously observed in Figure 1d that the device array presents good conformability, and can seamlessly attach onto the human skin. It is worth mentioning that the device array is extremely soft so that it can follow the movement of the human wrist without delamination phenomenon. These results fully testify that this reliable strategy enables the design of sophisticated and high‐resolution soft devices and circuits.

### 2.2 Full‐Photolithographic Conformable OTFTs

**Figure 2:**
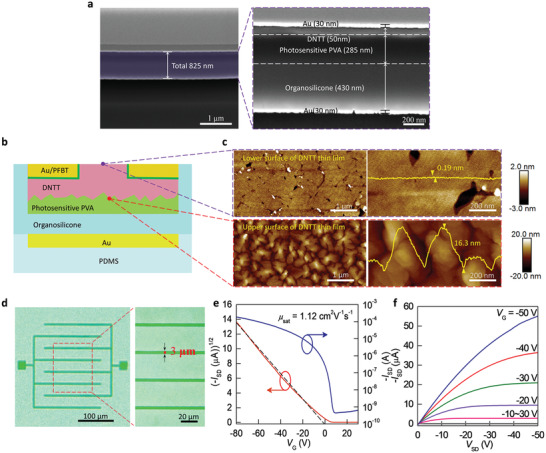
Electrical performance of full‐photolithographic conformable organic transistors. a) Cross‐sectional SEM images of a whole device. b) Structure schematic diagram of a transistor. The device is a bottom‐gate, top‐contact (BGTC) configuration with DNTT as the active layer. c) Atomic force microscope (AFM) images of the upper and lower surfaces of DNTT semiconductor layer. d) Optical microscopic image and its magnified view of a typical device with the electrode width as low as 3 µm. e,f) Typical transfer and output characteristics of the high‐precision device.

Based on our strategy, we can successfully fabricate full‐photolithographic conformable OTFTs. The thickness of the overall functional device is only 825 nm as shown in **Figure** **2**a. The cross‐sectional scanning electron microscopy (SEM) image clearly shows the thickness of each component. In addition, it can be clearly observed that each layer is tightly connected without delamination phenomenon. Figure 2b displays the schematic diagram of the structure of a device. The device is a BGTC configuration with dinaphtho[2,3‐b:2′,3′‐*f* ]thieno[3,2‐*b*]thiophene (DNTT) as the active layer. It is worth mentioning that we use the upper surface of the deposited semiconductor as the channel layer to achieve the top‐contact configuration for ensuring the device with good field‐effect performance. Different from the reported bottom‐contact OTFTs,^[^ ^24^, ^25^ ^]^ our top‐contact counterparts can effectively improve the carrier injection efficiency due to their larger charge injection area (Figure S5, Supporting Information). Figure 2c shows the morphology of the upper and lower surfaces of the DNTT semiconductor. Although the upper surface of the semiconductor is rougher than the lower surface, the device still shows excellently electrical performance. Figure 2d presents the optical microscopic images of a typical device. Thanks to the introduction of photolithography technology, the device has a high precision with the electrode width as low as 3 µm. Considering that 3 µm is the highest precision of our photolithography machine, it can be predicted that the device precision is much higher than 3 µm. Such a precision is difficult to achieve with other fabrication techniques, such as inkjet printing, screen printing, and shadow mask.^[^ ^26^, ^27^, ^28^ ^]^ The transfer and output characteristics of the device are displayed in Figure 2e,f, respectively. The channel length and channel width are 30 and 1050 µm, respectively. The transistor shows the clear p‐type characteristics with well‐defined linear and saturation regimes. Figure 2f shows the evolution of *I*_SD_ with drain–source voltage (*V*_SD_), where each curve is measured at a different *V*_G_, and demonstrates linearity at low *V*_SD_ and a clear transition from the linear to saturation regime. The linear behaviors at low *V*_SD_ suggest good electrode contact.^[^ ^29^ ^]^ This device exhibits a remarkable field‐effect mobility in the saturation regime of *μ*_sat_ = 1.12 cm^2^ V^−1^ s^−1^ and a near‐zero threshold voltage of *V*_T_ = −0.51 V. The comparable mobility and threshold voltage of full‐photolithographic conformable OTFTs to the commonly used hydrogenated amorphous silicon (a‐Si:H) show the strong potential of this strategy in broad applications, for example, in active matrix liquid crystal displays (AMLCDs).^[^ ^30^ ^]^

The excellent electrical properties of our full‐photolithographic conformable OTFTs can be attributed to the following three aspects: i) the top‐contact geometry. So far, almost all the reported photolithographic flexible OTFTs are bottom‐gate, bottom‐contact geometries, and exhibit low field‐effect performance (Table S1, Supporting Information). Despite the bottom‐gate, bottom‐contact OTFTs can avoid solvent and radiation damages to organic semiconductors caused by the photolithography process, they inevitably suffer from a tiny fraction of the gate‐field‐induced carrier injection, resulting in poor field‐effect performance. In contrast, our bottom‐gate, top‐contact OTFTs have a larger carrier injection area and ensure efficient carrier injection,^[^ ^31^ ^]^ which improves the field‐effect performance. ii) Functionalization of electrodes: to improve the morphology of the deposited organic semiconductor thin films on electrodes, we applied 2,3,4,5,6‐pentafluorobenzenethiol (PFBT) self‐assembled monolayers (SAMs) to modify the source–drain electrodes. Figure S6 (Supporting Information) displays the device diagrams and the corresponding transfer characteristics of full‐photolithographic conformable OTFTs with and without PFBT SAMs’ modification. After the functionalization of source–drain electrodes, the mobility is enhanced by two orders of magnitude (Figure S6a_2_,b_2_, Supporting Information). iii) Optimization of dielectric layer: in our experiments, we used photosensitive PVA and elastic organosilicone as the dielectric layer. The double dielectric layer is employed not only for organic semiconductor photolithography, but also for improving the electrical performance of the full‐photolithographic OTFTs. Figure S7 (Supporting Information) shows the transfer and output characteristics with and without photosensitive PVA layer. By adding the photosensitive PVA layer, mobility increased by two times compared to a single organosilicone‐based device, due to the elimination of thermal expansion damage of the organic semiconductor caused by the elastic dielectric layer (Figure S8, Supporting Information). As shown in Figure S8 (Supporting Information), after adding photosensitive PVA, the on‐state current of the device is significantly increased in the same test range, and the threshold voltage is decreased. This indicates that the increased mobility of photosensitive PVA/organosilicone‐based device is not due to the thickening of the insulation layer. Therefore, our full‐photolithographic conformable OTFTs exhibit outstanding field‐effect performance.

### 2.3 Device Array

**Figure 3:**
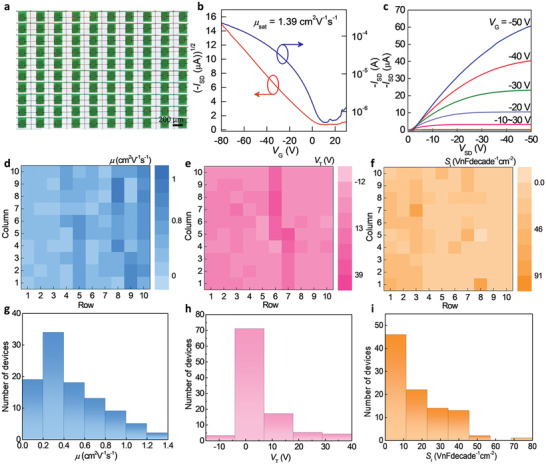
Uniformity test of 10 × 10 full‐photolithographic conformable organic transistor array. a) Transmission optical microscopy image of a typical DNTT transistor array with the device number of 100. b,c) Typical transfer and output characteristics of the transistor array. d–f) Field‐effect mobility (*μ*), threshold voltage (*V*_T_), and normalized subthreshold swing (*S_i_*) color maps and g–i) distribution of the 100 transistors.

As organic electronic devices mature into practical applications, the success rate of device fabrication and performance distribution become the crucial factors. Here, we have fabricated a DNTT full‐photolithographic conformable OTFT array of 100 transistors. Its photo images are shown in **Figure** **3**a. Compared to the other reported flexible transistor array which is achieved by print or shadow‐mask technology,^[^ ^23^, ^31^, ^32^, ^33^, ^34^ ^]^ we successfully integrated modern photolithography technology into our OFET fabrication, and then the integration level and pattern flexibility of our devices are effectively improved. Figure 3b,c shows transfer and output curves for DNTT OTFTs. All transistors performed well, and showed quite clear p‐type characteristics, with well‐defined saturation and linear regimes. The linear behavior at a low *V*_SD_ in the output curve suggested good electrode contact (Figure 3c). According to the transfer curve, mobility was extracted in the saturated regime. Figure 3d–i shows color maps and the corresponding distributions of the calculated field‐effect characteristic parameters in the DNTT full‐photolithographic conformable OTFT array. As shown in Figure 3d–f, device uniformity is nicely expressed by the spatial distribution of the transistor performance. The statistical results of Figure 3g–i show the mobility, threshold voltage (*V*_T_), and normalized subthreshold swing (*S*_i_) distribution, respectively. The high yield, 100% working transistor array shows the mobility distribution where 98% of transistors have mobilities higher than 0.1 cm^2^ V^−1^ s^−1^. The mobility average value is 0.47 cm^2^ V^−1^ s^−1^ with a standard deviation of 0.31 cm^2^ V^−1^ s^−1^. The highest mobility reaches 1.39 cm^2^ V^−1^ s^−1^ in the saturation regime. This value is superior to all reported full‐photolithographic OTFTs (Table S1, Supporting Information). About 60% of transistors show the threshold voltages in the range from −5 to 5 V. The lowest threshold voltage is at −0.04 V. About 97% of transistors display *S_i_* lower than 50 VnF decade^−1^ cm^−2^. It is worth mentioning that our device can be operating normally under relatively low voltage (Figure S9, Supporting Information), demonstrating the potential application in future electronic skin.

Though some novel studies on photolithographic OTFTs have been reported, none has led to fully photolithographic conformable OTFT array. Most of them can only achieve partially photolithographic OTFTs, that is, only one component of organic transistor such as electrode, semiconductor, or dielectrics is patterned by photolithography, and others are patterned by shadow masks or other techniques. This severely limits the miniaturization and precision of devices, and then inevitably results in low device density and low integration.^[^ ^14^ ^]^ So far, the reported conformable OTFT array with the highest level of integration is 347 transistors cm^−2^ by partially photolithographic dielectric.^[^ ^4^ ^]^ Based on the conventional layer‐by‐layer photolithography process, several studies can construct fully photolithographic BGBC OTFTs on rigid inorganic or flexible plastic substrates,^[^ ^14^, ^35^, ^36^, ^37^ ^]^ but these arrays are not conformable and requires specific organic semiconductor materials. In addition, most of them are the bottom‐gate, bottom‐contact geometries, thereby, show the low mobilities. Altogether these results demonstrate that our photolithography‐compatible strategy provides an effective way to fabricate high‐performance top‐contact transistor array, in favor of developing the engineering of complex and multifunctional micro/nanoscale organic electronic devices.

### 2.4 Conformability of Devices

**Figure 4:**
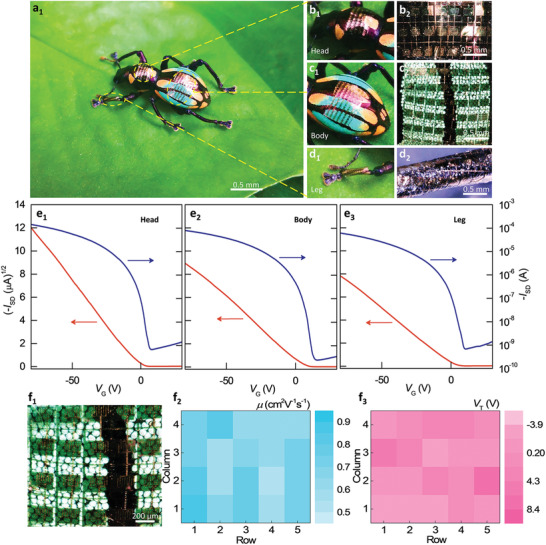
Full‐photolithographic conformable transistor array for wearable electronics. a_1_–d_1_) Digital photos and b_2_–d_2_) 3D optical microscope images of transistor arrays conforming on different parts of a beetle specimen. e_1_–e_3_) Typical transfer characteristics of transistor arrays on different parts of the beetle specimen, such as head, body, and leg. f_1_–f_3_) 3D optical microscope image and field‐effect mobility (*µ*) and threshold voltage (*V*_T_) color maps of a transistor array with the device number of 20.

To examine the conformal and wearable capability of the fabricated full‐photolithographic OTFTs, we attached them to different parts of a real beetle, including the head, body, and leg, and measured their field‐effect performances (**Figure** **4**). It can be clearly observed in Figure 4 a_1_–d_2_ that the device array can intimately adhere to the beetle surface, presenting outstanding conformability. Figure 4 e_1_–e_3_ shows the typical transistor characteristics of the real devices on different parts. On the head, body, and leg, the calculated mobilities are 0.94, 0.59, and 0.38 cm^2^ V^−1^ s^−1^, respectively. They show a threshold voltage at −1.53, 4.18, and 0.67 V, respectively. The normalized subthreshold swing *S_i_* is as low as 4.1, 3.63, 6 VnF decade^−1^ cm^−2^. To further confirm the good conformability of the DNTT full‐photolithographic conformable OTFT array, we measured electrical properties of the 4 × 5 OTFT array onto the body of the beetle (Figure 4 f_1_). Figure 4 f_2_,f_3_ depicts the corresponding spatial distribution of the field‐effect performance. All the devices can normally operate and maintain the typical p‐type field‐effect performance. The average mobility is 0.68 cm^2^ V^−1^ s^−1^ with a standard deviation of 0.12 cm^2^ V^−1^ s^−1^. The average *V*_T_ value is 0.71 V with a standard deviation of 3.17 V. In addition, our devices can operate normally under both tensile and compressive stresses (Figure S10, Supporting Information), and under different humid environments (Figure S11, Supporting Information). The device can operate normally even at the relative humidity (RH) as high as 90.4%. Our fabricated devices show outstanding transistor characteristics on the curved objects, indicating the promising potential for next‐generation soft wearable electronics.

### 2.5 Universality of Our Strategy

**Figure 5:**
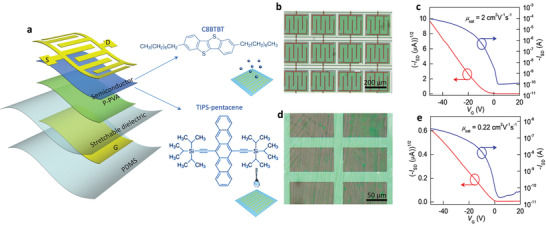
Universality of the scalable photolithography strategy. a) Device schematic and molecular structures of C8‐BTBT and TIPS‐pentacene. This strategy is compatible with different organic semiconductor. C‐8BTBT thin film and TIPS‐pentacene single‐crystal array were prepared by vacuum depositing (top inset) and drop casting (bottom inset), respectively. b–e) Transmission optical microscopy image and the corresponding transfer characteristics of photolithographic conformable C8‐BTBT and TIPS‐pentacene transistors.

To illustrate the capabilities of our strategy for different organic semiconductor materials, we selected two typical commercial materials including ultrathin 2,7‐dioctyl[1]benzothieno[3,2‐*b*][1]benzothiophene (C8‐BTBT) and 6,13‐bis(triisopropylsilylethynyl)‐pentacene (TIPS‐pentacene) as active layers, and measured their field‐effect performance. C8‐BTBT thin film was fabricated by vapor‐processed thermal evaporation, while TIPS‐pentacene microwire array was prepared by solution‐processed drop coating as shown in **Figure** **5**a. Figure 5b–e shows transmission optical microscopy images and the corresponding transfer characteristics of photolithographic conformable C8‐BTBT and TIPS‐pentacene OTFTs, respectively. All OTFTs were tested in the atmosphere, and all show typical p‐type characteristics with excellent field‐effect performance. According to the transfer curve in Figure 5c, the calculated mobility of C8‐BTBT OTFTs is as high as 2 cm^2^ V^−1^ s^−1^. This value is higher than those of all the reported photolithographic OTFTs (Table S1, Supporting Information). The mobility of the TIPS‐pentacene single‐crystal OTFT is up to 0.22 cm^2^ V^−1^ s^−1^ (Figure 5e). Although previously reported several photolithographic strategies,^[^ ^14^, ^29^, ^38^ ^]^ such as orthogonal photolithography, photosensitizer doping, and semiconductor molecular design, have been developed to fabricate photolithographic OTFTs, these strategies require specific semiconductor materials to prepare these photolithographic organic devices, which are difficult to achieve universal and widespread application in the future industrialization and commercial production. On the contrary, our fabrication approach is not only applicable to various organic semiconductor materials with different morphology or variety classes, but also compatible with organic semiconductors in liquid‐phase or gas‐phase preparation processes. Our strategy offers a universal platform for constructing full‐photolithographic high‐performance OTFTs, and demonstrates a great prospect of application in the development of organic devices toward industrialized flexible and elastic electronic products.

## 3 Conclusions

In summary, we have developed a facile photolithography strategy for achieving high‐performance top‐contact conformable OTFTs. The general fabrication process flow does not cause serious damage on organic semiconductors by utilizing the photosensitive PVA as protection and dielectric layers, and is designed to enable high integration, high precision, high device yield, device‐to‐device uniformity, good material compatibility between layers, and good electrical and mechanical performances. More impressively, the obtained conformable OTFT exhibits an unprecedented device density of 1523 transistors cm^−2^, and the mobility is as high as 1–2 cm^2^ V^−1^ s^−1^. These key values are superior to the previously reported all the photolithographic OTFTs. Additionally, the fabricated devices show excellent conformability. Their flexible and elastic nature allows them to well adhere onto various curved surfaces without wrinkles, and cracks, showing its versatility and near‐universal applicability. Our strategy offers a general and facile route to incorporate photolithography technology into organic electronics, and presents potential for developing skin‐like soft electronic devices with high device density and high performance.

## 4 Experimental Section

##### Materials

All processing materials were purchased from commercial sources and used as received. Organic semiconductors, including DNTT (99%), C8‐BTBT (99%), and TIPS‐pentacene (99%), were purchased from Sigma–Aldrich. OTS (95%) and PFBT (95%) were obtained from Acros and TCI (Shanghai) Development Co., Ltd, respectively. PVA (*M*_w_ ≈ 205 000 g mol^−1^) was supplied by Sigma–Aldrich, and ammonium dichromate (≥99%) serving as the photosensitizer was purchased from Sinopharm Chemical Reagent Co., Ltd. The elastic materials used in this study, including Dow Corning 1–257(DC 1–2577) and Dow Corning Sylgard 184 (DCS 184 or usually called PDMS), were obtained from Dow Corning Corporation. The solvent of the elastic materials was OS‐20 (Dow Corning Corporation).

##### Fabrication of the Fully Photolithographic Conformable OTFT Array

i) Si wafers, modified by a self‐assembled layer of OTS (Acros, 95%), were employed for the carrier chip during the experiments. The Si wafers were hydroxylated with an O_2_ plasma treatment (100 W, 30 s), and then loaded into a vacuum oven for the OTS vapor treatment (60 °C, 0.5 h). Hereafter, the undiluted PDMS (DCS 184) was dropped onto the OTS‐modified Si wafers. After curing, the PDMS layer was peeled off with tweezers, and then an ultrasmooth OTS monolayer was formed on the Si wafers. ii) The 30 nm thick Au micropatterns severing as source–drain electrodes were prepared on OTS‐modified Si wafers by a lift‐off photolithography process. The photoresist used in these experiments was AZ5200NJ (Clariant). After removing photoresist, the sample was immerged into PFBT solution (PFBT:toluene = 1:1000 by volume) for 2 min at room temperature. iii) Organic semiconductor (DNTT, C8‐BTBT, or TIPS‐pentacene) and photosensitive PVA (PVA:deionized water:ammonium dichromate = 105:2000:2 by weight) were deposited successively on PFBT‐modified Au source–drain electrodes (Figure 1a ‐I). Subsequently, the photosensitive PVA was photopatterned by exposure under ultraviolet light (wavelength = 365 nm) for 1.5 min to initiate the photo‐crosslinking reaction in selective areas defined by a photolithographic mask (Figure 1a ‐II). After this, deionized water was used to dissolve the unexposed areas of photosensitive PVA, with the exposed areas preserved. In order to fully crosslink these preserved areas, the sample was further baked at 60 °C for 10 min in an oven. iv) Based on the protection of the photopatterned photosensitive PVA, the organic semiconductor was etched by oxygen plasma for forming photolithographic micropatterns (Figure 1a ‐III). DNTT was thermally evaporated at a rate of ≈0.1 Å s^−1^ under 5 × 10^−4^ Pa with a substrate temperature of 60 °C, while C8‐BTBT was deposited at a rate of ≈0.20 Å s^−1^ under 4 × 10^−4^ Pa with a substrate temperature of 50 °C. TIPS‐pentacene microwire array was formed via drop casting a droplet of TIPS‐pentacene/chloroform solution onto the prefabricated PFBT‐modified Au source–drain electrodes. The thickness (*d*) of the photopatterned photosensitive PVA was 400 nm. v) In order to obtain thin and uniform elastic dielectric layer, organosilicone DC 1–2577 were dissolved in OS‐20 (DC 1–2577:OS‐20 = 1:5 by volume), followed by spin coating (6000 rpm, 60 s) onto the micropatterned sample (Figure 1a ‐IV). The annealing temperature was set at 60 °C for 0.5 h. In these experiments, both the photopatterned photosensitive PVA and the elastic DC 1–2577 act as the dielectric layer. The measured capacitance (*C_i_*) was 1.5 nF cm^−2^. In addition, organosilicone DC 1–2577 can be patterned via photolithography and reactive‐ion etching for further perforation. vi) The elastic dielectric and photolithographic gate electrode embedded in PDMS were laminated together by plasma oxidation and heating (Figure 1a ‐V). The gate electrode embedded in PDMS was obtained by peeling it off from an OTS‐modified Si wafer. The elastic dielectric and the gate electrodes embedded in PDMS were, respectively, placed in a plasma oxidation chamber and oxidized for 100 s, aligned, and then heated in the oven at 60 °C for 10 min. vii) By peeling off the whole structure (source–drain electrodes/organic semiconductor/photosensitive PVA/DC 1–2577/gate electrode/PDMS support layer) from OTS/Si wafer (Figure 1a ‐VI), the fully photolithographic conformable organic transistor array was obtained (Figure 1a ‐VII). The electrode was flipped over to form bottom‐gate top‐contact conformable OTFT array. The thickness of the entire device was 10 µm.

##### Characterization

The 3D optical images were obtained using a 3D digital microscope (Keyence, VHX‐5000). AFM measurements were carried out on a Dimension Icon instrument using a NanoScopeV9 controller (Bruker, Inc.). The electrical characteristics of OTFT devices were recorded with a Keithley 4200 SCS and a Cascade M150 probe station in a clean and shielded box at room temperature in air. All the field‐effect parameters were calculated with the standard equation in the saturation regime. The standard equation is $\mu =\frac{2L}{WC_{i}}{\left(\frac{\sqrt[{\partial }]{I_{\mathrm{DS}}}}{\partial VG}\right)}^{2}$, where *C_i_* is the dielectric capacitance, *W* is the channel width, and *L* is the channel length. All experiments were performed in compliance with the relevant laws and institutional guidelines, and Northeast Normal University confirmed that formal approval was not necessary for this study; the only participant in this study was one of the co‐authors.

##### Statistical Analysis

The data used for the extraction of field‐effect parameters such as *μ* and *V*_T_ were not preprocessed before the analysis. Device outliers were excluded prior to analysis based on the reliability of the *I*_SD_^1/2^ versus *V*_G_ curve. Results were presented as (mean ± SD), where SD represents the standard deviation, followed by the appropriate units. All experiments had a sample size of at least *n* = 20, and statistical analysis was performed using OriginPro 9.0 (OriginLab Corporation).

## Conflict of Interest

The authors declare no conflict of interest.

## Supporting information

Supporting Information
