# Supplementary material for: High‐Performance Full‐Photolithographic Top‐Contact Conformable Organic Transistors for Soft Electronics
Source: Adv Sci (Weinh). 2021 Feb 18;8(9):2004050. doi: 10.1002/advs.202004050 (PMC8097323; doi:10.1002/advs.202004050)
Supplement: Supplementary file 1 — Supporting Information [file ADVS-8-2004050-s001.pdf]

© 2021 Wiley-VCH GmbH

ADVANCED SCIENCE
Open Access

Supporting Information

for *Adv. Sci.,* DOI: 10.1002/advs.202004050

High-Performance Full-Photolithographic Top-Contact Conformable Organic Transistors for Soft Electronics

Xiaoli Zhao, Shuya Wang, Yanping Ni, Yanhong Tong, Qingxin Tang, and Yichun Liu*

Copyright WILEY-VCH Verlag GmbH & Co. KGaA, 69469 Weinheim, Germany, 2013.

Supporting Information

High-Performance Full-Photolithographic Top-Contact Conformable Organic Transistors for Soft Electronics

Xiaoli Zhao, Shuya Wang, Yanping Ni, Yanhong Tong, Qingxin Tang, and Yichun Liu*

Center for Advanced Optoelectronic Functional Materials Research, and Key Lab of UV-Emitting Materials and Technology of Ministry of Education, Northeast Normal University, 5268 Renmin Street, Changchun 130024, China

E-mail: tangqx@nenu.edu.cn

Tel./fax: +86-431-85099873.

Table S1. Detailed performance list for the reported photolithographic organic thin-film transistors.

Photolithography	Photolithographic component	Mechanical property	Semiconductor	μ ($cm^2V^{-1}s^{-1}$)	Device configuration	Ref.
Partial	S/D /G electrode	Flexible	PTV	3×10^{-4}	TGBC	[1]
Partial	S/D electrode; semiconductor	Rigid	P3HT	5×10^{-4}	BGBC	[2]
Partial	Semiconductor	Rigid	P3HT	0.03	BGTC	[3]
Partial	S/D electrode	Flexible	Pentacene	0.1	BGBC	[4]
Partial	S/D electrode	Rigid	P(NDI2OD-T2)	0.11	TGBC	[5]
Partial	S/D electrode	Rigid	C60	0.11	BGTC	[6]
Partial	S/D/G electrode	Conformable	DNTT	0.13	TGBC	[7]
Partial	S/D electrode	Rigid	Pentacene	0.17	TGBC	[8]
Partial	S/D electrode	Rigid	Pentacene	0.17	BGBC	[9]
Partial	Dielectric	Rigid	Pentacene	0.25	BGTC	[10]
Partial	S/D electrode	Rigid	Pentacene	0.29	BGTC	[11]
Partial	S/D electrode	Rigid	Pentacene	0.3	BGBC	[12]
Partial	S/D electrode	Flexible	Pentacene	0.36	BGBC	[13]
Partial	Dielectric	Rigid	PTDPPTFT4	0.45	BGTC	[14]
Partial	S/D/G electrode	Flexible	Pentacene	0.55	BGBC	[15]
Partial	S/D electrode	Rigid	DNTT	0.77	BGBC	[16]
Partial	Dielectric	Conformable	CONPHINE	1.11	TGBC	[17]
Partial	Semiconductor	Rigid	PTCDI-C13	1.65	BGBC	[18]
Partial	G electrode	Conformable	C_8-BTBT	2	BGTC	[19]
Partial	S/D electrode	Rigid	C10 -DNBDT	2.5	TGBC	[8]
Partial	S/D/G electrode	Conformable	C_8-BTBT	2.7	BGTC	[20]
Partial	S/D electrode	Rigid	C10 -DNTT	5	TGBC	[8]
Full	S/D/G electrode; semiconductor; dielectric	Rigid	P2TDC13FT4	0.1	BGTC	[21]
Full	S/D/G electrode; semiconductor; dielectric	Rigid	Ph-PXX	0.1	BGBC	[22]
Full	S/D/G electrode; semiconductor	Flexible	PTDPPSe-SiC4	0.35	BGBC	[23]
Full	S/D/G electrode; semiconductor; dielectric	Flexible	P(DPP2DT-TVT)	1.03	TGBC	[24]
Full	S/D/G electrode; semiconductor; dielectric	Conformable	DNTT<br>C8-BTBT	1.39<br>2	BGTC	This work

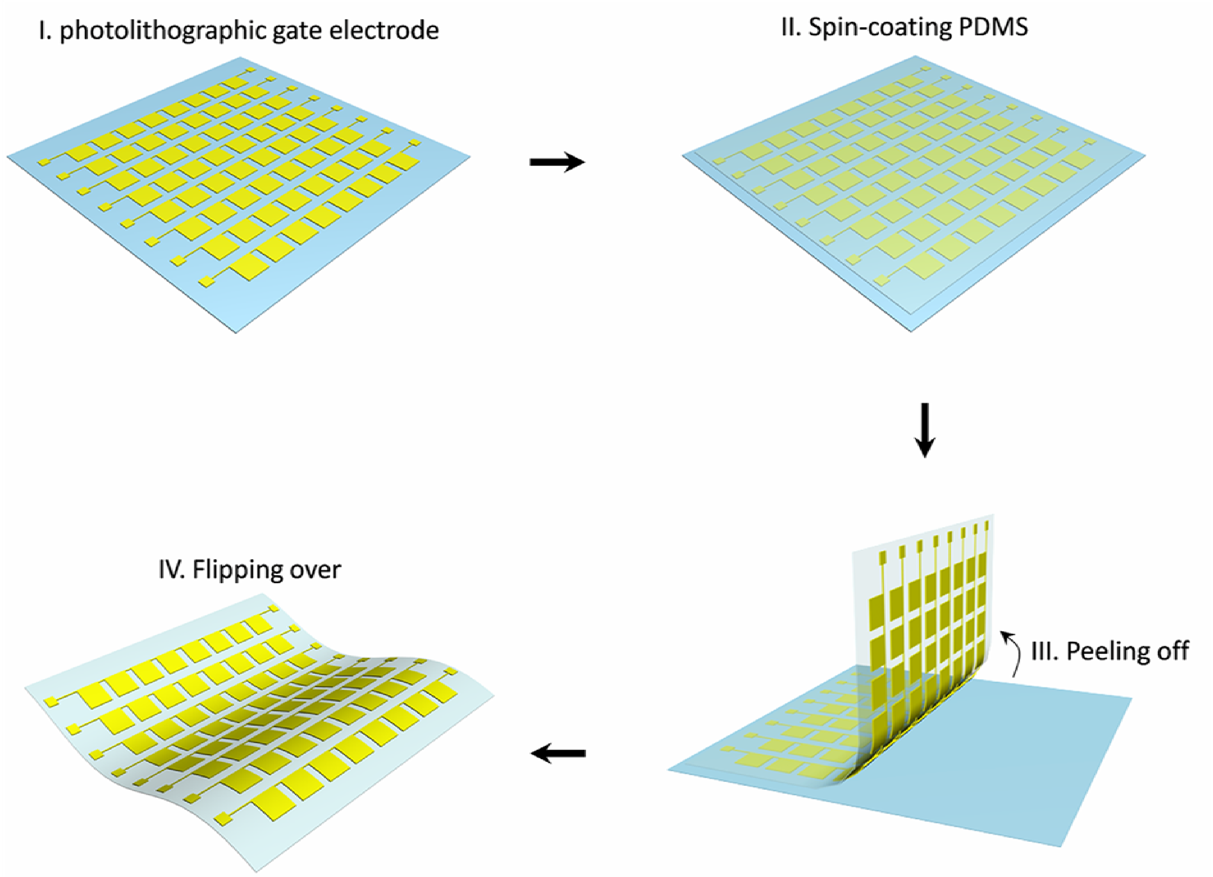

Figure S1. Schematic diagram of the preparation process of the elastic embedded gate electrode. Initially, Au patterns were prepared on OTS/Si substrate by photolithography. Then PDMS was spin coated on Au patterns. After the PDMS was cured, the electrode was peeled from the OTS/Si substrate. Finally, a flexible and elastic embedded gate electrode was obtained.

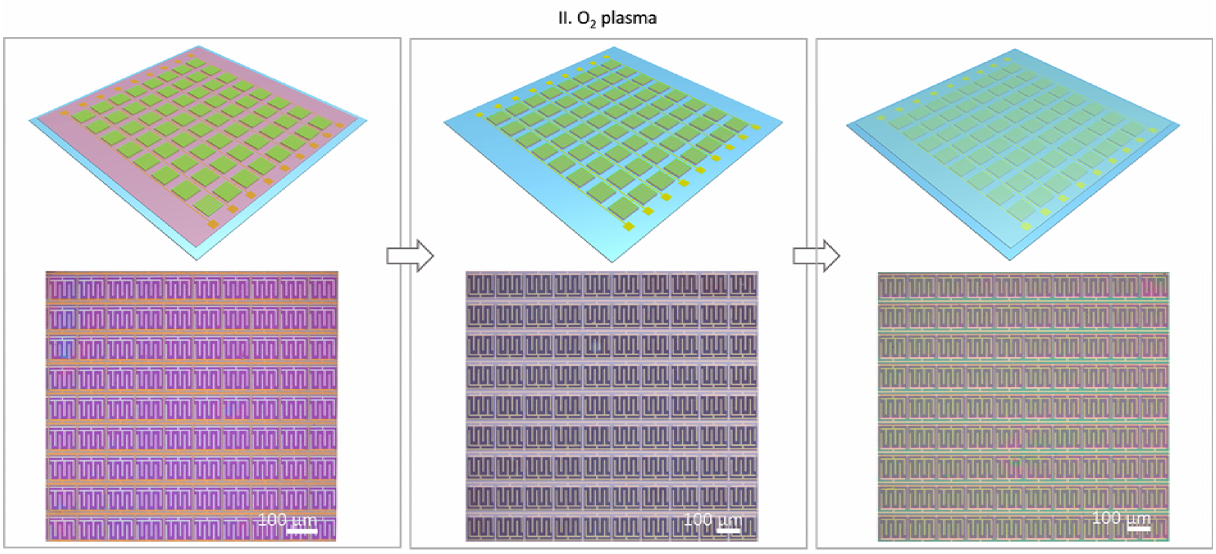

Figure S2. The preparation process of the photolithographic DNTT organic semiconductor. Initially, photosensitive PVA occurred crosslinking reaction under UV irradiation and formed fine patterns after developing in water. Next, based on the protection of photo-crosslinked PVA, the patterned organic semiconductor was precisely defined by oxygen plasma etching. After that, elastic organosilicone was spin-coated onto the patterned sample as the dielectric layer and adhesive layer. The corresponding optical microscope images clearly show the photolithographic process of the organic semiconductor.

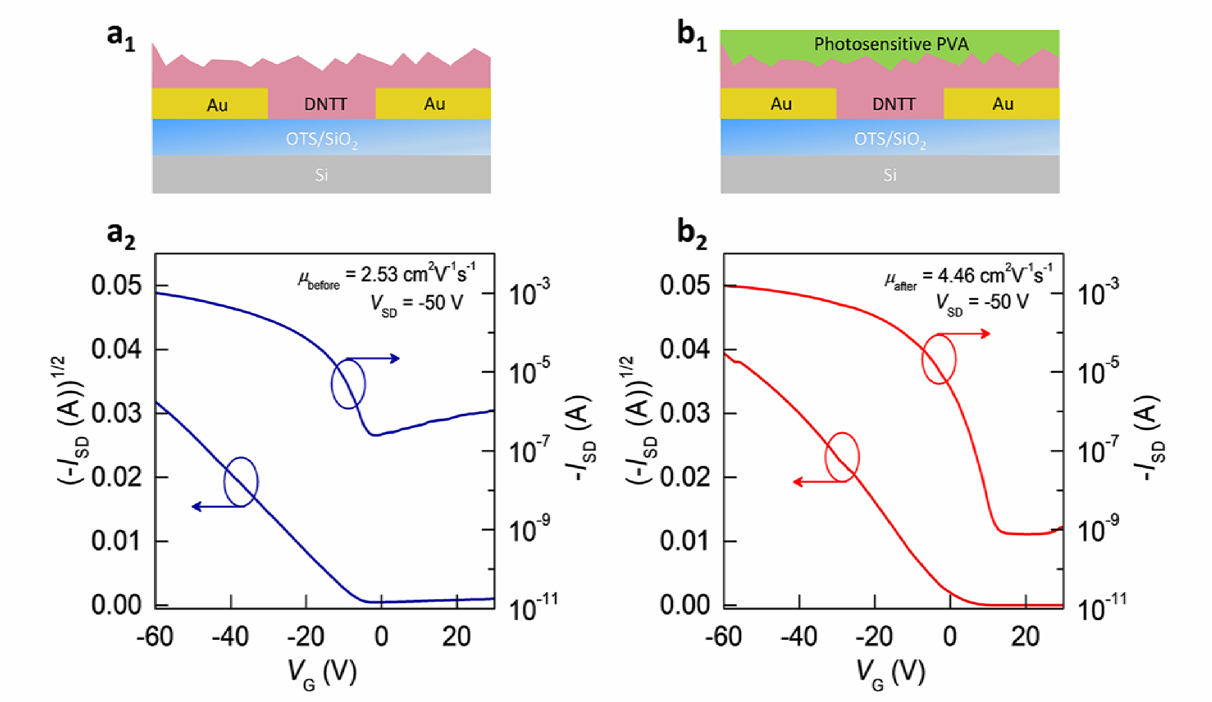

Figure S3. Schematic diagram and transfer characteristics of DNTT OTFTs with and without the photo-crosslinked PVA. After the semiconductor was patterned, the electrical properties of the transistor have not decayed, or even increased. Because photosensitive PVA is water soluble and extremely mild without any damage to the organic semiconductor.

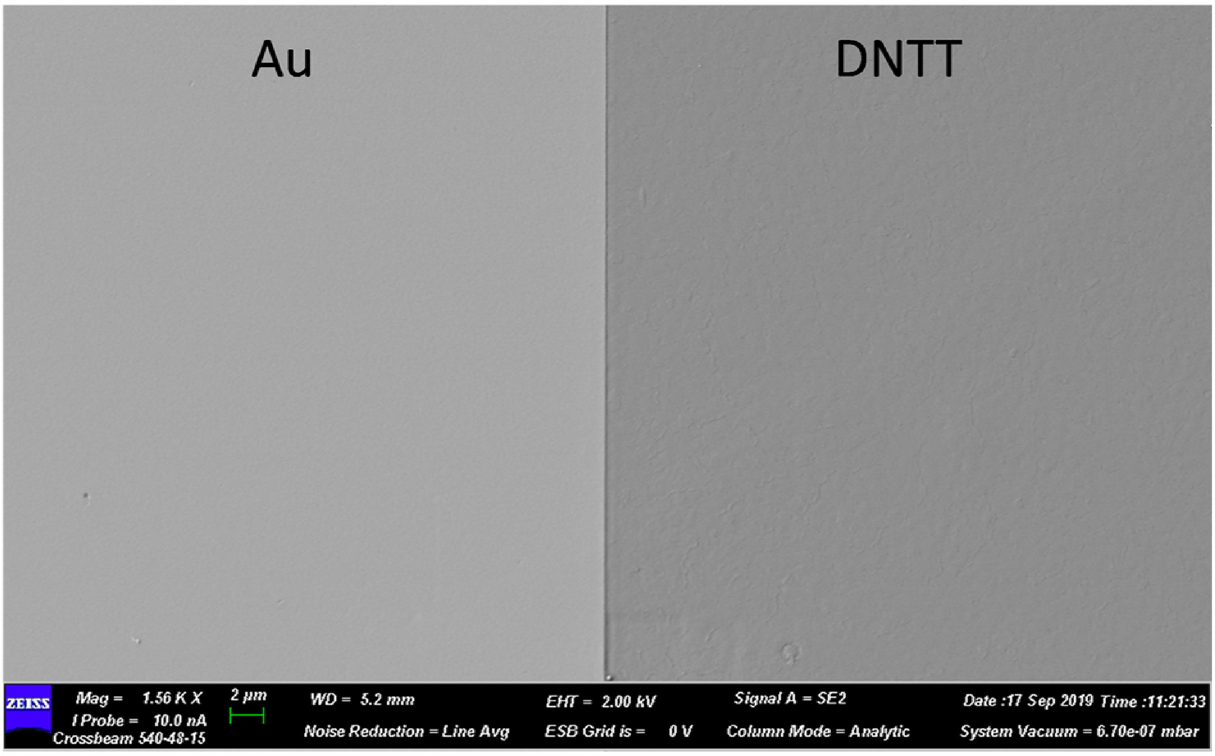

Figure S4. A SEM image of a full-photolithographic conformable OTFT. It can be clearly seen that the electrode is well-defined with a sharp edge, and there are no residues in void areas.

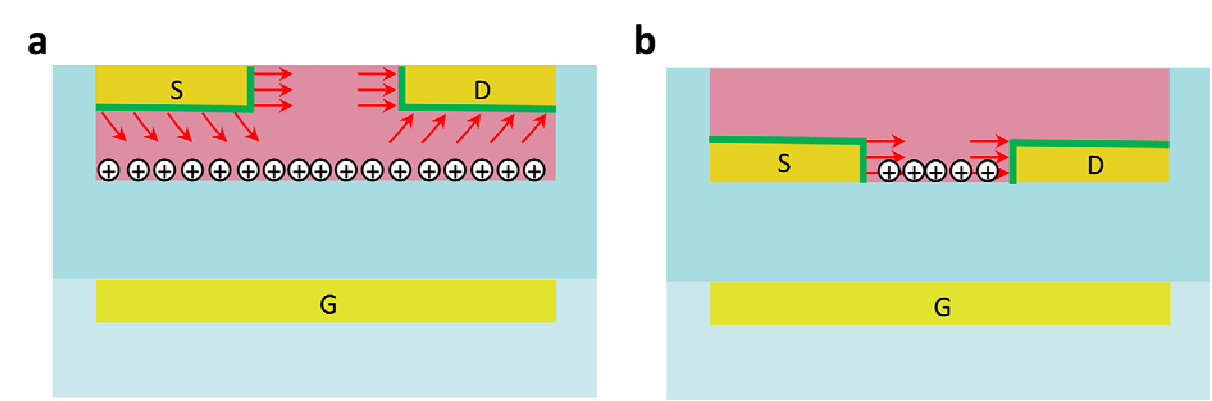

Figure S5. Schematic images of transistor devices based on different architectures. (a) bottom-gate top-contact geometry. (b) Bottom-gate Bottom -contact geometry. Compared to a bottom-contact geometry, top-contact counterpart has a larger charge injection area, which ensures high electrical properties.

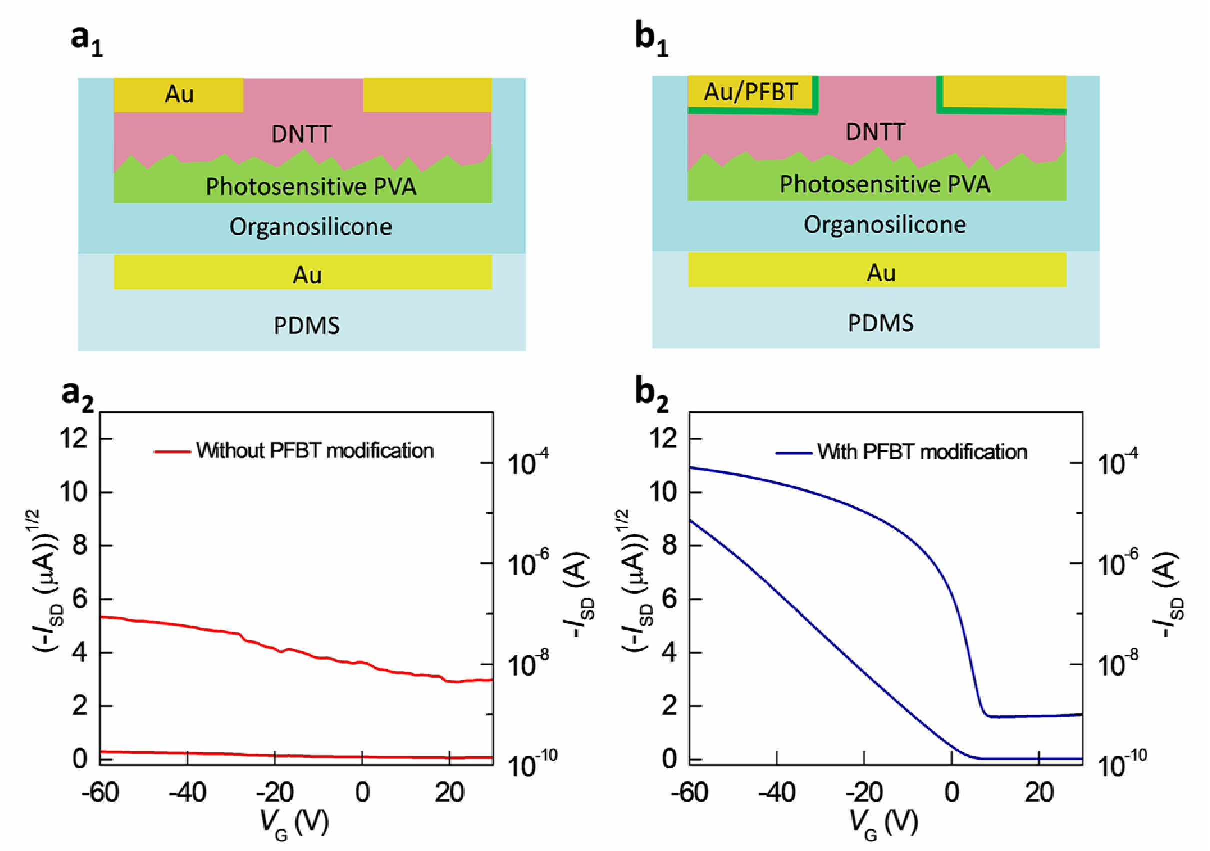

Figure S6. Schematic diagram and transfer characteristics of DNTT full-photolithographic conformable OTFTs with and without the PFBT SAMs modification. After the functionalization of source-drain electrodes, the mobility is enhanced by two orders of magnitude.

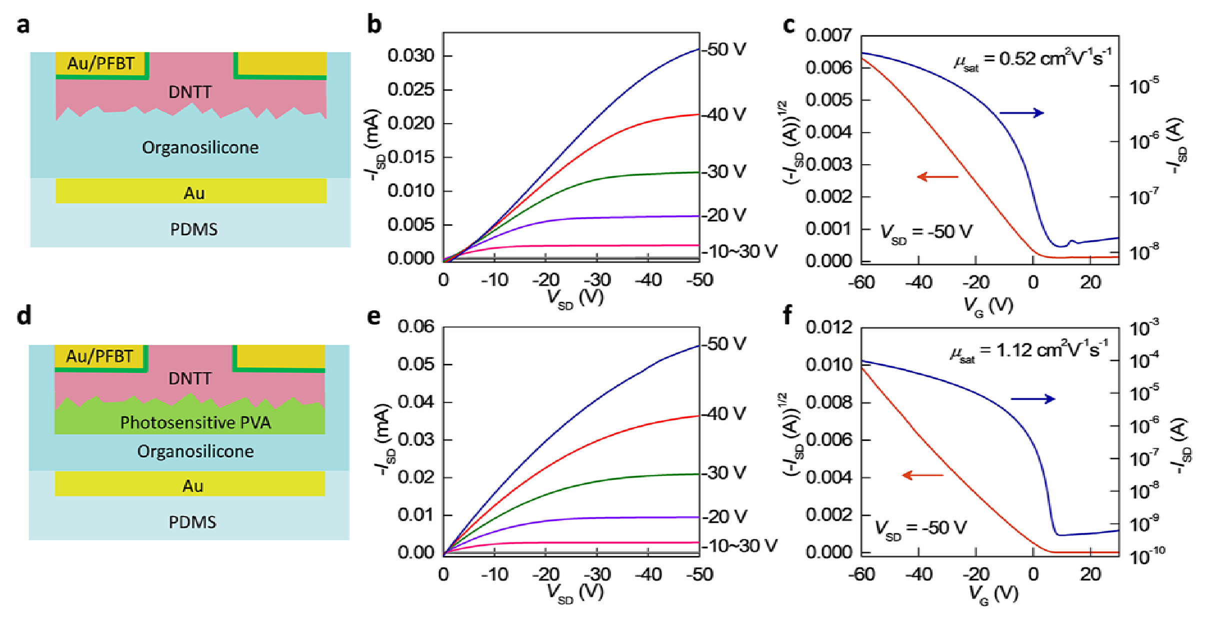

Figure S7. Schematic diagram, and typical transfer and output characteristics of DNTT full-photolithographic conformable OTFTs with and without photosensitive PVA layer. By adding the photosensitive PVA layer, mobility increased by 2 times compared to a single organosilicone-based device.

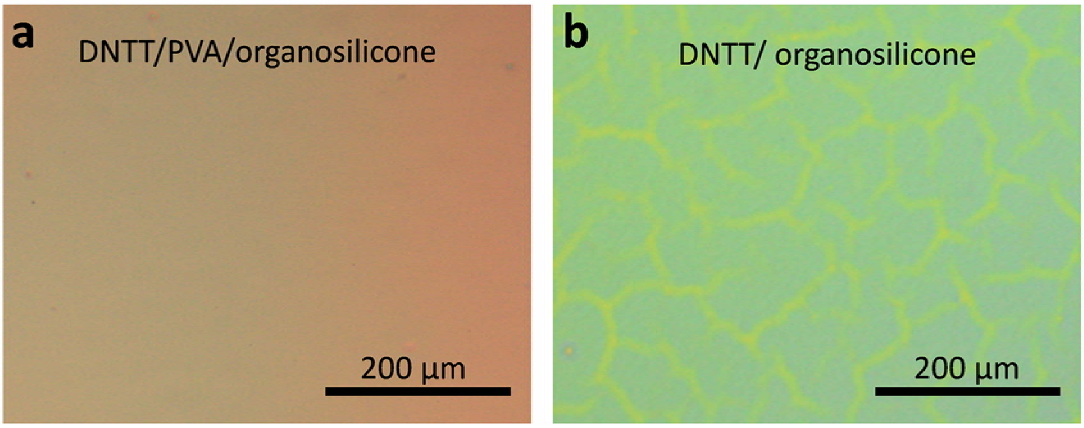

Figure S8. Optical microscope images of DNTT/PVA/organosilicone and DNTT/ organosilicone. By adding the photosensitive PVA layer, thermal expansion damage of the organic semiconductor caused by the elastic dielectric layer can be avoided.

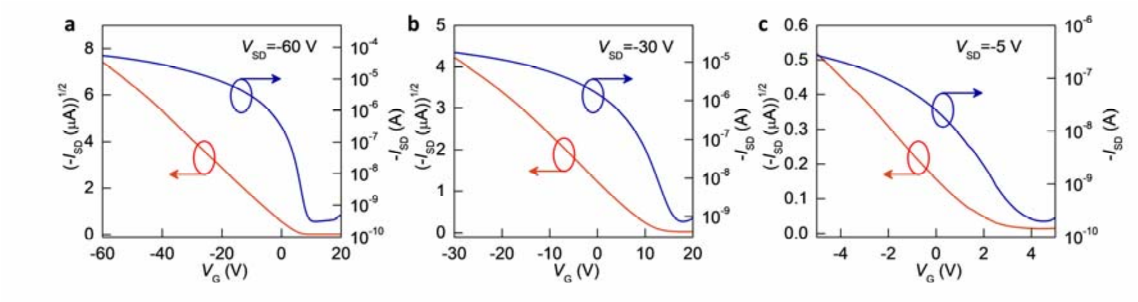

Figure S9. Transfer characteristics of full-photolithographic conformable transistors at operating voltage: a) V_{SD}=-60 V; V_{SD}=-30 V; V_{SD}=-5 V.

At different V_{SD} of -60, -30, -5 V, mobility is 0.66, 0.61, 0.27 $cm^2V^{-1}s^{-1}$, respectively. The device can be operating normally at a relatively low voltage, due to using both high-*k* photo-crosslinked PVA and low-*k* organicsilicone materials as the dielectric layer.

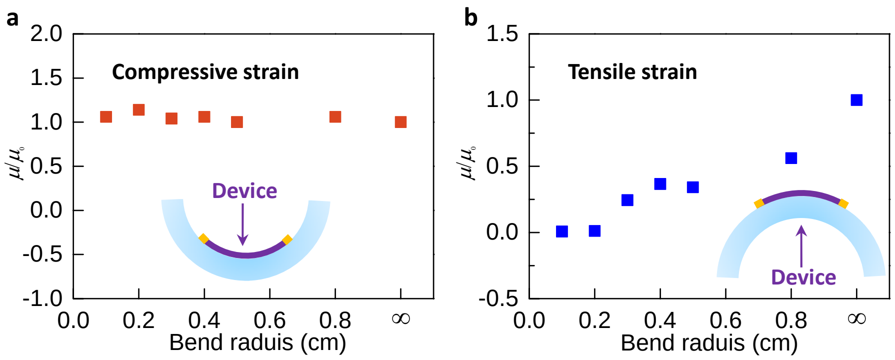

Figure S10. Dependence of mobility changes on the bending radius under different state. (a) Compressive strain, (b) Tensile strain.

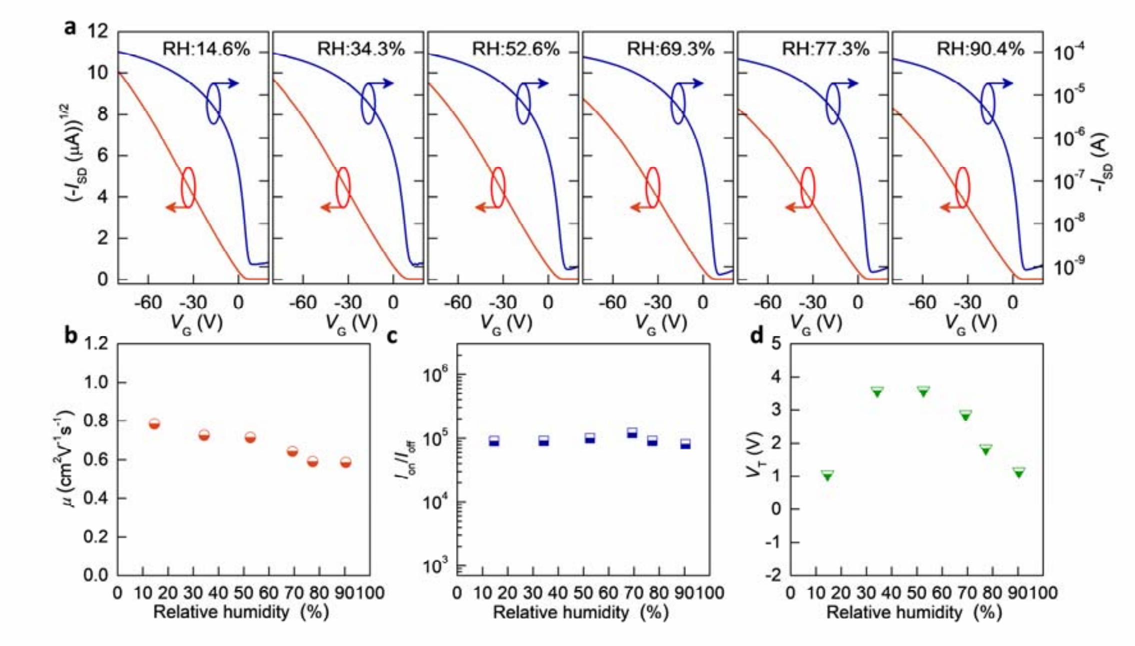

Figure S11. a) Transfer characteristics of full-photolithographic conformable transistors at different relative humidity. b) Dependence of μ, I_{on}/I_{off}, and V_T on the relative humidity.

The device can operate normally even at the RH as high as 90.4%. As the relative humidity increases from 14.6 to 90.4%, the mobility (μ) of the conformable device gradually decreases from 0.78 to 0.59 $cm^2V^{-1}s^{-1}$. The current on/off ratio (I_{on}/I_{off}) is almost unchanged ～10^5 under different RH. The threshold voltage (V_T) varies from 1.06 to 1.15 V.

Supporting Information References

[1] C. J. Drury, C. M. J. Mutsaers, C. M. Hart, M. Matters, D. M. de Leeuw, *Appl. Phys. Lett.* **1998**, *73*, 108.

[2] J. R. Chan, X. Q. Huang, A. M. Song, *J. Appl. Phys.* **2006**, *99*, 023710.

[3] C. Balocco, L. A. Majewski, A. M. Song, *Org. Electron.* **2006**, *7*, 500.

[4] J. Jang, Y. Song, H. Oh, D. Yoo, D. Kim, H. Lee, S. Hong, J.-K. Lee, T. Lee, *Appl. Phys. Lett.* **2014**, *104*, 053301.

[5] K. J. Baeg, D. Khim, S. W. Jung, M. Kang, I. K. You, D. Y. Kim, A. Facchetti, Y. Y. Noh, *Adv. Mater.* **2012**, *24*, 5433.

[6] H. Kleemann, A. A. Zakhidov, M. Anderson, T. Menke, K. Leo, B. Lüssem, *Org. Electron.* **2012**, *13*, 506.

[7] A. Reuveny, S. Lee, T. Yokota, H. Fuketa, C. M. Siket, S. Lee, T. Sekitani, T. Sakurai, S. Bauer, T. Someya, *Adv. Mater.* **2016**, *28*, 3298.

[8] K. Nakayama, M. Uno, T. Uemura, N. Namba, Y. Kanaoka, T. Kato, M. Katayama, C. Mitsui, T. Okamoto, J. Takeya, *Adv. Mater. Interfaces* **2014**, *1*, 1300124.

[9] A. Petritz, M. Krammer, E. Sauter, M. Gärtner, G. Nascimbeni, B. Schrode, A. Fian, H. Gold, A. Cojocaru, E. Karner-Petritz, R. Resel, A. Terfort, E. Zojer, M. Zharnikov, K. Zojer, B. Stadlober, *Adv. Funct. Mater.* **2018**, *28*, 1804462.

[10] J. Jang, S. H. Kim, J. Hwang, S. Nam, C. Yang, D. S. Chung, C. E. Park, *Appl. Phys. Lett.* **2009**, *95*, 073302.

[11] C.-L. Fan, W.-C. Lin, C.-C. Lee, Y.-Z. Lin, B.-R. Huang, *Jpn. J. Appl. Phys.* **2016**, *55*, 026502.

[12] L. Li, L. Jiang, W. Wang, C. Du, H. Fuchs, W. Hu, L. Chi, *Adv. Mater.* **2012**, *24*, 2159.

[13] D. Ji, L. Jiang, H. Dong, Q. Meng, Z. Wang, H. Zhang, W. Hu, *ACS Appl. Mater.*

Interfaces **2013**, *5*, 2316.

[14] Q. Shi, Y. Xie, S. Cai, W.-Y. Lee, Z. Bao, J. R. Matthews, K. L. Simonton, T. E. Myers, R. A. Bellman, M. He, H. H. Fong, *Org. Electron.* **2014**, *15*, 991.

[15] D. Ji, L. Jiang, X. Cai, H. Dong, Q. Meng, G. Tian, D. Wu, J. Li, W. Hu, *Org. Electron.* **2013**, *14*, 2528.

[16] S. Liu, A. Al-Shadeedi, V. Kaphle, C.-M. Keum, B. Lüssem, *Org. Electron.* **2017**, *45*, 124.

[17] S. Wang, J. Xu, W. Wang, G. N. Wang, R. Rastak, F. Molina-Lopez, J. W. Chung, S. Niu, V. R. Feig, J. Lopez, T. Lei, S. K. Kwon, Y. Kim, A. M. Foudeh, A. Ehrlich, A. Gasperini, Y. Yun, B. Murmann, J. B. Tok, Z. Bao, *Nature* **2018**, *555*, 83.

[18] A. G. Ismail, *Org. Electron.* **2018**, *56*, 111.

[19] N. Cui, H. Ren, Q. Tang, X. Zhao, Y. Tong, W. Hu, Y. Liu, *Nanoscale* **2018**, *10*, 3613.

[20] N. Cui, Q. Tang, H. Ren, X. Zhao, Y. Tong, Y. Liu, *J. Mater. Chem. C* **2019**, *7*, 5385.

[21] A. A. Zakhidov, H. H. Fong, J. A. DeFranco, J.-K. Lee, P. G. Taylor, C. K. Ober, G. G. Malliaras, M. He, M. G. Kane, *Appl. Phys. Lett.* **2011**, *99*, 183308.

[22] T.-H. Ke, R. Müller, B. Kam, M. Rockele, A. Chasin, K. Myny, S. Steudel, W. D. Oosterbaan, L. Lutsen, J. Genoe, L. van Leuken, B. van der Putten, P. Heremans, *Org. Electron.* **2014**, *15*, 1229.

[23] E. K. Lee, C. H. Park, J. Lee, H. R. Lee, C. Yang, J. H. Oh, *Adv. Mater.* **2017**, *29*, 1605282.

[24] M. J. Kim, M. Lee, H. Min, S. Kim, J. Yang, H. Kweon, W. Lee, D. H. Kim, J. H. Choi, D. Y. Ryu, M. S. Kang, B. Kim, J. H. Cho, *Nat. Commun.* **2020**, *11*, 1520.
